# Ultrasound-guided radial vein cannulation for general anesthesia in cases with difficult peripheral venous access: a report of two cases

**DOI:** 10.1186/s40981-024-00743-y

**Published:** 2024-09-19

**Authors:** Hironori Motoyama, Joho Tokumine, Yukiko Saito, Kiyoshi Moriyama, Tomoko Yorozu

**Affiliations:** Department of Anesthesiology, Kyorin University School of Medicine, 6-20-2 Sinkawa, Mitaka, Tokyo, 181-8611 Japan

**Keywords:** Difficult peripheral venous access, Ultrasound-guided vascular access, Radial vein

## Abstract

**Background:**

Despite advancements in ultrasonography, locating peripheral veins for catheter placement remains a challenge in patients with altered anatomy owing to multiple surgeries. Herein, we highlight the potential of using the radial vein as an alternative site for ultrasound-guided peripheral venous catheterization.

**Case presentation:**

We present two cases of patients with extensive surgical histories, including multiple abdominal surgeries, leading to difficult peripheral venous access. Traditional sites for peripheral venous catheterization were unsuitable due to vein narrowing or lack of visibility. In both cases, ultrasonography helped identify the radial vein as the only viable site for catheter placement. The patients underwent successful ultrasonography-guided catheterization of the radial vein without complications, facilitating medical management, including anesthesia induction and intraoperative monitoring.

**Conclusions:**

The radial vein is a feasible and safe alternative for ultrasound-guided peripheral venous access in patients where traditional venous access sites are compromised.

## Background

Difficult peripheral venous cannulation occurs in 10–24% of adult patients [1], for whom conventional or peripherally inserted central venous catheters are typically utilized. Recent advancements in ultrasound guidance have significantly enhanced the success rates of peripheral venous cannulation, particularly among patients with challenging peripheral venous access [2].

While attempting radial artery catheterization, the radial vein may occasionally be cannulated inadvertently. To date, no complications following such accidental cannulation have been reported. We document two cases where peripheral venous catheters were successfully placed into the radial veins under ultrasound guidance in patients with challenging peripheral venous access.

## Case presentation

### Case 1

**Fig. 1:**
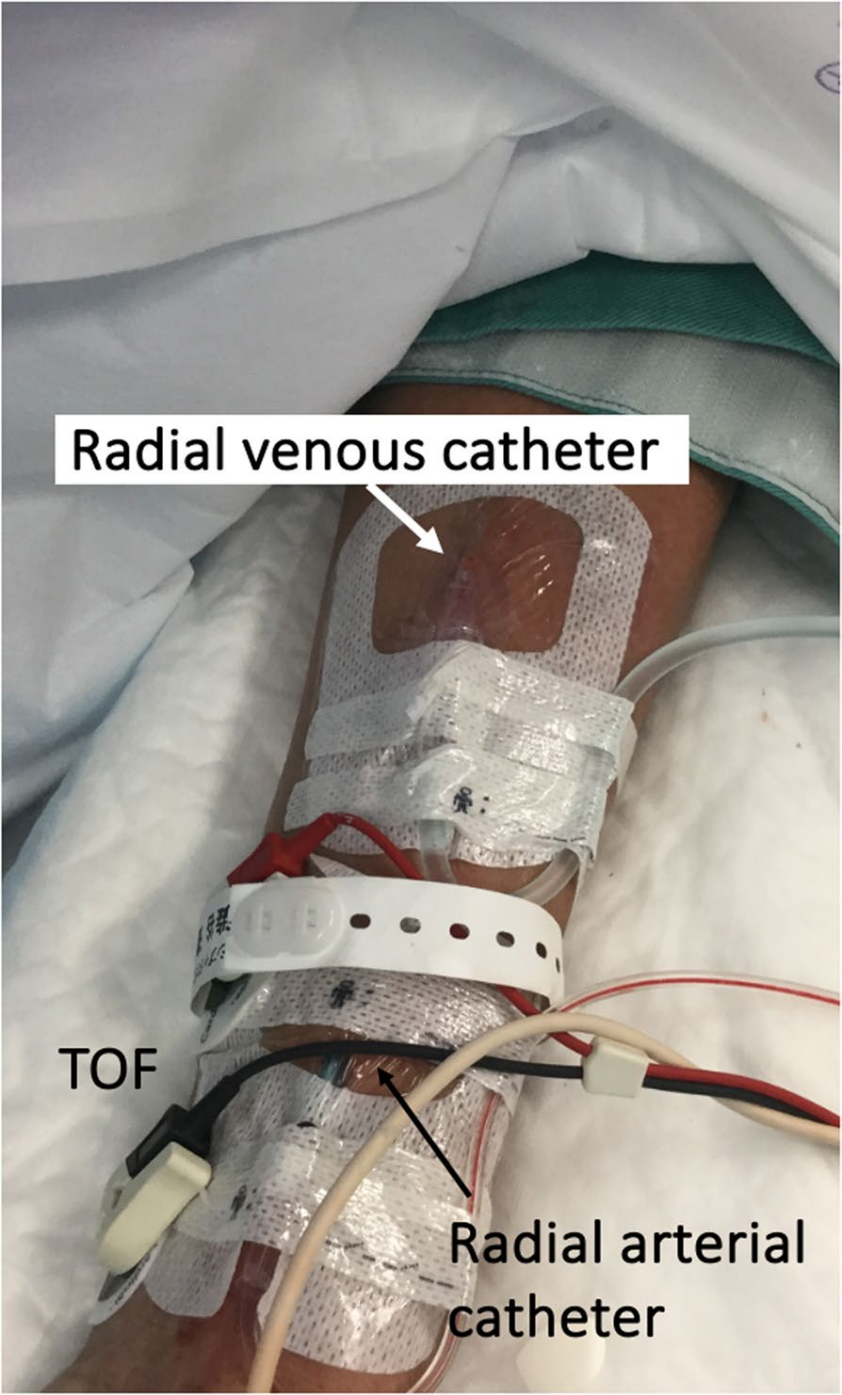
A radial venous catheter inserted in the left forearm in Case 1

**Fig. 2:**
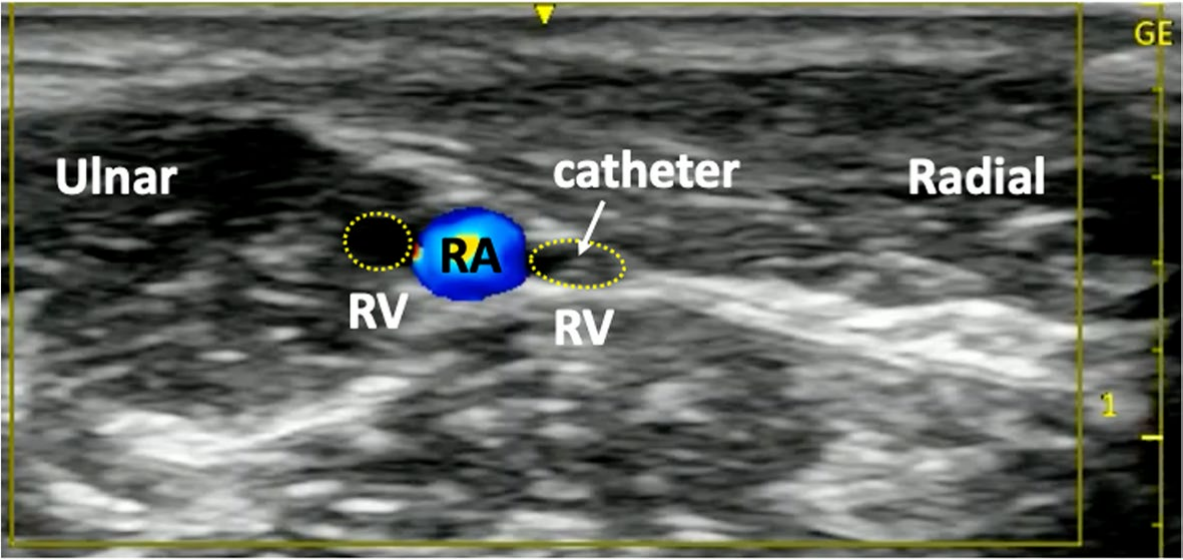
Ultrasound short-axis view of the left forearm in Case 1

A 70-year-old man (height, 168 cm; body weight, 54 kg) underwent total cystectomy with ileal conduit for bladder cancer and colostomy for pelvic abscess and small intestine perforation. Two years later, he developed a paralytic bowel obstruction requiring colostomy. A peripherally inserted central venous catheter (PICC; single lumen, 3 Fr.) was inserted in his right upper arm for total parenteral nutrition. On the day of the surgery, general anesthesia was induced via propofol administered from the PICC. An intraarterial pressure line was inserted into the left radial artery. Intraoperatively, the patient experienced a sudden blood loss of approximately 300 mL within 30 min owing to the surgical release of colon adhesions from previous surgeries; therefore, an additional venous catheter for potential transfusion was required. Despite the extensive ultrasonographic examinations, no peripheral veins were visible. The cubital veins, including median cubital, cephalic, and basilic veins, were either narrowed or not visible. However, the radial veins in the left forearm were accessible, and a long intravenous catheter (external diameter 20 G, catheter length, 51 mm; Surflo™ I.V. Catheter, TERMO Co. Japan) was inserted under ultrasonographic guidance (Figs. 1 and 2). Successful hemostasis resulted in a final blood loss limited to 300 mL, rendering intraoperative transfusion unnecessary.

### Case 2

A 65-year-old man (height, 170 cm; body weight, 88 kg) with recurrent nerve palsy post-aortic replacement surgery for dissecting aortic aneurysm was scheduled for laryngoplasty. His medical history included psychostimulant-induced psychosis and hepatitis C virus infection. The patient was apprehensive about repeated attempts at peripheral venous cannulation for the induction of general anesthesia. During preoperative consultation, the anesthesiologist advised that central venous catheter placement might be necessary in case peripheral access was challenging. The patient opted against central venous catheter placement owing to discomfort experienced during prior surgeries. Consequently, the anesthesiologist agreed to pursue peripheral venous access.

**Fig. 3:**
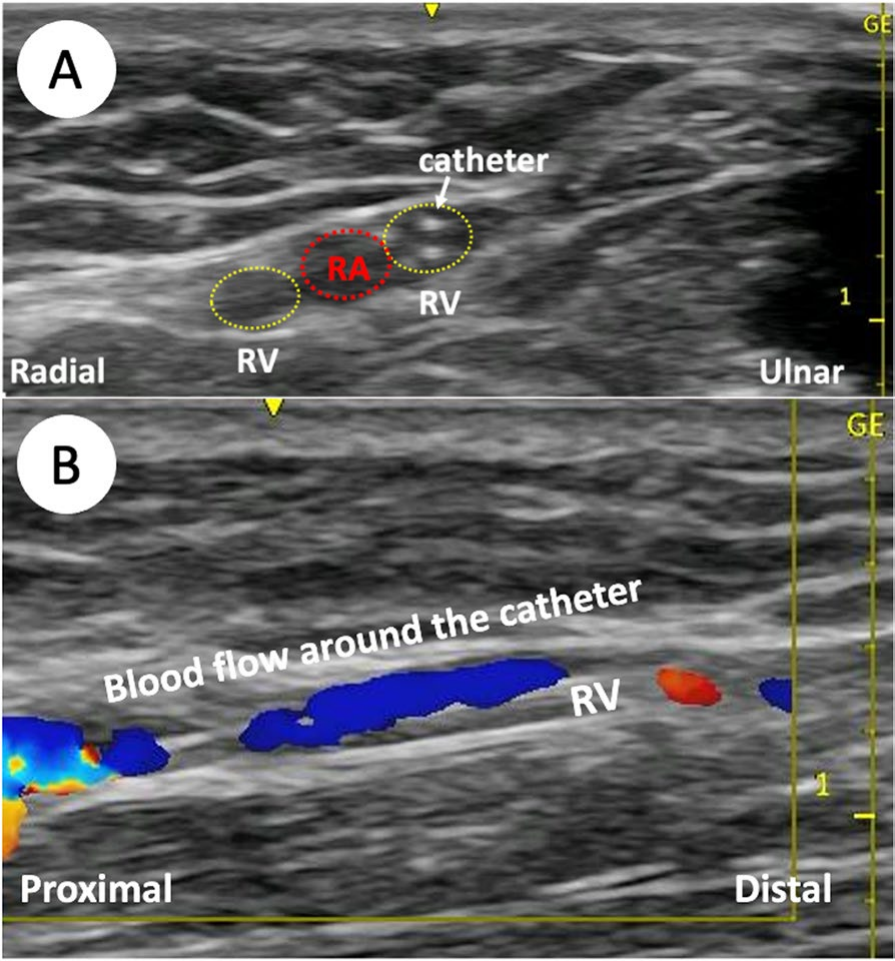
Ultrasound short- (**A**) and long-axis (**B**) view of the right forearm in Case 2

On the day of surgery, despite the absence of visible superficial peripheral veins in the forearm, radial veins were detected in the right forearm. After injecting 1% lidocaine (0.5 mL) with a 25-G hypodermic needle, a long intravenous catheter (20 G, inserting length 51 mm, Surflo™ I.V. Catheter, TERMO Co. Japan) was subsequently placed in the radial vein under ultrasonographic guidance (Fig. 3). Propofol was administered through this catheter, and general anesthesia was induced. No complications were reported with perioperative catheter maintenance, and the patient expressed satisfaction during the postoperative visit.

## Discussion

Antecubital cephalic or median veins are typically preferred for emergent peripheral venous access owing to their ease of access [3]. However, repeated venous access can cause narrowing or obstruction of these veins. Consequently, catheter placement in these veins is often unsuccessful in patients with difficult venous access. Systematic, stepwise approaches and a broad repertoire of techniques are essential for managing such cases [4]. Ultrasound guidance is instrumental in facilitating peripheral venous catheter placement in patients with difficult venous access [5]. The midline catheter has also gained attention as a safe and long-indwelling ultrasound-guided peripheral venous catheter for patients with difficult venous access [6]. In Case 2, a midline catheter was a feasible alternative. However, its placement requires a sterilized environment, including using sterilized surgical gloves and drapes, and the arm of the patient must be abducted during the procedure, which complicates insertion during surgery under general anesthesia, as evidenced in Case 1.

Witting et al. reported higher success rates of cannulation for venous diameters > 4 mm than for < 4 mm and venous depths of 3–15 mm versus < 3 or > 15 mm [7]. In Case 1, the venous diameter was 1 mm, and the venous depth was 6 mm. In Case 2, the venous diameter was 2 mm, and the venous depth was 7 mm. While the diameters in these two cases were narrow, the depths were within the ranges for ultrasound puncture. If the needle is inserted shallower than 3 mm, ultrasound focusing is insufficient, and lateral resolution is poor, resulting in poor image quality. In addition, the needle should strike the center of the vein as soon as it is advanced subcutaneously. However, if the needle is deflected, the lateral wall of the vein will be damaged and cannulation will not be possible due to hemorrhage. Therefore, ultrasound-guided venous cannulation requires a needle to be inserted to a depth of at least 3 mm. In contrast, if the needle is inserted too deep, advancing the needle into the target vein becomes difficult. Concerning the optimal depth of the vein, no precise reference can be found because there are no other reports on cannulation of the radial vein.

Radial veins are a pair of veins that accompany the radial artery. To our knowledge, this is the first case report of targeting the radial vein for cannulation. Therefore, the optimal site or technique for radial vein cannulation is not conclusive. However, because the radial vein always accompanies the radial artery, the optimal conditions for cannulation of the radial artery to the radial vein are expected to be extrapolated. Wu et al. reported that the success rate of ultrasound-guided radial artery cannulation was higher in the mid-forearm than in the wrist [8]. Yücel et al. also reported higher success rates in the distal quarter of the forearm than in the mid-forearm [9]. The radial nerve to the radial artery and veins in the proximal quarter are closely located. Therefore, the radial vein cannulation in this region carries a risk of nerve injury. In summary, the optimal cannulation site of the radial vein may be the distal quarter of the forearm, whereas the proximal from the mid-forearm and wrist are less likely to be suitable for cannulation. The most important technical aspect of cannulation would be the entry angle of the needle into the vein. If the entry angle is too steep, the posterior wall of the vein is easily damaged during the cannulation. In our experience, the best entry angle for cannulation is a shallow angle of less than 30°. As in ultrasound-guided radial artery cannulation, the needle is gradually advanced into the target vein while confirming its position with ultrasound [10, 11]. Once the needle tip reaches the vein, further decreasing the entry angle is recommended to achieve cannulation. The shallower the needle entry angle, the greater the distance from the sticking site to the target vein. Therefore, we use relatively long needles (usually approximately 5 cm) for deeper veins such as the radial vein.

We demonstrated that the radial vein is a viable target for ultrasound-guided peripheral venous access in patients with difficult peripheral venous access and the cannulation can be performed safely with ultrasound guidance.

## Data Availability

Not applicable.

## Abbreviation

PICC: Peripherally inserted central catheter
